# Distribution and Ecological Traits of *Cotoneaster integerrimus* in South Korea

**DOI:** 10.3390/biology14121737

**Published:** 2025-12-04

**Authors:** Gyeong-Yeon Lee, Deokki Kim, Seung-Eun Lee, Tae-Bok Ryu

**Affiliations:** Research Center for Endangered Species, National Institute of Ecology, Yeongyang 36561, Republic of Korea; ky5724@nie.re.kr (G.-Y.L.); ecose2543@nie.re.kr (S.-E.L.)

**Keywords:** *Cotoneaster integerrimus*, species distribution model (SDM), ecological divergence, geographical peripheral population (GPP), glacial relict, limestone habitat

## Abstract

Although the rare plant *Cotoneaster integerrimus* is distributed across Eurasia, ecological information on its isolated populations at the easternmost range limit in Korea has been entirely lacking. This study was conducted to (1) characterize the environmental characteristics of the habitat of Korean *C. integerrimus* populations and (2) predict potential habitats via a simple species distribution model (SDM) based on ridge logistic regression and presence–background data, providing a foundation for effective conservation strategies. To this end, we analyzed habitat type, topography, and light conditions through field surveys and combined these data with an SDM fitted to six known occurrences on limestone ridges. Results revealed a clear ecological divergence; the Korean population is biased toward partial shade and north-facing slopes within the forest understory, in contrast to European populations inhabiting open, rocky sites. This distribution pattern is interpreted as a local adaptive strategy that reduces exposure to hot and humid summer conditions. Furthermore, a unique morphological trait not reported in European populations was identified: dense persistent hairs that remain until seed maturity. The SDM analysis showed moderate discrimination (training AUC = 0.784) and indicated that high elevation and ridge topography (Topographical Position Index, TPI) acted as key habitat factors, whereas annual mean temperature was the strongest limiting factor. Mapping the upper decile (top 10%) of predicted suitability within the limestone belt highlighted a small, spatially restricted set of high-elevation ridges as candidate microrefugia and survey priorities. This study suggests that the Korean *C. integerrimus* population may have undergone local adaptation due to isolation. Furthermore, this population is considered both a Geographical Peripheral Population (GPP) and a glacial relict, and is assessed to be vulnerable to climate change. Given that the SDM is based on only six occurrences and shows variable performance among spatial folds, all spatial predictions and variable effects should be regarded as exploratory and spatially conservative rather than as definitive habitat projections. These findings, therefore, support the urgent need to establish in situ and ex situ conservation strategies that preserve this geographically peripheral population as an irreplaceable component of the species’ genetic diversity.

## Simple Summary

This study investigates the isolated population of *Cotoneaster integerrimus* in Korea, which represents the easternmost limit of its distribution. Unlike European populations that typically grow in sunny, rocky areas, the Korean population is found in shady, cool forest understories and retains a densely hairy calyx (or unique hairs on its fruits). These distinct characteristics suggest that the population may have undergone local adaptation to survive the region’s hot summers. As a likely glacial relict, this population is vulnerable to climate change, emphasizing the urgent need for conservation measures to protect its unique morphological and ecological characteristics.

## 1. Introduction

The genus Cotoneaster (Rosaceae), comprising small trees or deciduous shrubs, is distributed across temperate regions of Eurasia and North Africa [1,2,3]. *Cotoneaster integerrimus*, a deciduous shrub native to East Asia and Europe [4,5], exhibits high adaptability to diverse climatic and topographic conditions [6,7]. This species serves as a key indicator for shrub communities inhabiting dry, highly alkaline rocky environments [8]. It is also recognized as a representative constituent of the ‘Rock pear scrub’ habitat type under the EUNIS (European Union Nature Information System) classification [9]. Within Europe, its distribution ranges from rocky areas to open forest edges, and it is considered ecologically important for biodiversity conservation due to its unique habitat preferences and environmental adaptability. Recent studies have reported physiological changes in this species associated with climate change [10] and habitat loss driven by land-use changes and human activities [8]. Accordingly, understanding the ecological distribution and habitat characteristics of native species such as *C. integerrimus* has become a critical task in conservation biology and ecogeography [11,12].

The distribution of *Cotoneaster integerrimus* has been reported across extensive regions beyond Central Europe, including Iran [13], and East Asian countries such as China [14], India [15], and North Korea [16,17]. However, in stark contrast to its widely recognized distribution, in-depth research on East Asian populations remains scarce. Although several phylogenetic studies of Cotoneaster species in East Asia have been conducted [18], they have primarily focused on macro-level taxonomic relationships within the genus. Consequently, fundamental ecological baseline data for *C. integerrimus*—such as detailed distribution patterns, population sizes, and environmental requirements—remain limited. This research gap poses a significant constraint to the development of effective conservation and management strategies for *C. integerrimus* in East Asia.

In parallel, species distribution models (SDMs) have become an important tool for studying rare or data-poor plant species and for guiding conservation planning. Recent SDM applications have been used to predict habitat suitability for the endangered medicinal plant *Saussurea medusa* and to identify potential refugia under future climate scenarios [19]. Similarly, SDMs for rare endemic Myrtaceae in the Brazilian Atlantic Forest have highlighted the difficulty of conserving “invisible species” known from very few localities [20]. The distribution of the rare, endemic, tertiary relict *Syringa josikaea* in the Western Romanian Carpathians has been modeled to locate suitable habitats and support protection of relict populations [21]. For the genus Cotoneaster, recent modeling of *C. multiflorus* under current and future climate conditions has provided new insight into niche characteristics and potential range shifts [22]. Together, these studies show that carefully designed and cautiously interpreted SDMs can offer useful, preliminary guidance for survey design and conservation of narrow-range woody species, even when occurrence data are limited.

Against this background, the present study targets South Korea, which represents the easternmost distribution limit of *C. integerrimus*. Our objectives are: (1) to identify the actual distribution and habitat characteristics of Korean *C. integerrimus* populations through field surveys; (2) to obtain a first approximation of potential suitable habitats using a simple, ridge-based SDM fitted to presence–background data. The results are expected to improve understanding of the ecological characteristics of populations isolated at the eastern edge of the Eurasian continent and to provide baseline information for climate-change adaptation, conservation planning, and future biodiversity policies.

## 2. Materials and Methods

### 2.1. Field Survey and Site Characterization

**Figure 1:**
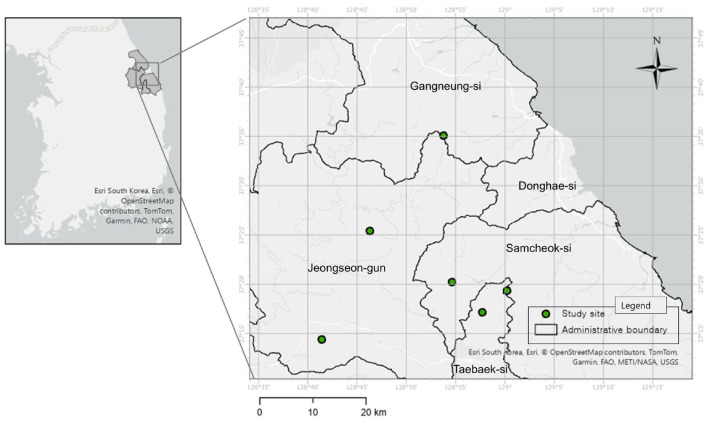
Study sites of *Cotoneaster integerrimus* in Korea.

This study was conducted to characterize the habitats of *Cotoneaster integerrimus*, a rare plant distributed in Korea. Study sites (n = 6, Figure 1) were selected focusing on major native habitats identified through existing literature reports and field surveys. Field surveys were intensively conducted from May to August 2025, during the active vegetation growth period. At the center of the *C. integerrimus* populations at each study site, the following environmental factors were measured:

- Habitat Type: Classified based on field observations of dominant overstory species and forest floor structure.
- Topographical Factors: To assess the topographical characteristics of each plot, altitude (m), slope angle, and aspect were measured using a smartphone-based app (calibrated before use). Slope angle (°) was measured as the maximum angle of the slope where the population was located. Aspect was recorded as the direction the slope faces (0–360°).
- Light Conditions: Recorded based on canopy openness and daily direct sunlight exposure, classified as follows:
  ✓ Full sun: Open sites (e.g., rocky debris slopes) receiving >6 h of direct sunlight without canopy obstruction.
  ✓ Partial shade: Sites where light is filtered by the overstory or receiving 3–6 h of partial direct sunlight (e.g., forest understory).

The field survey data were used to analyze the relationship between the distribution characteristics of *C. integerrimus* and habitat environmental factors. Data on habitat type, altitude, aspect, slope, and light conditions were synthesized to interpret the ecological niche of the Korean *C. integerrimus* populations.

### 2.2. Species Distribution Modeling (SDM)

Species Distribution Modeling (SDM) was used to investigate the habitat specificity and potential distribution of *C. integerrimus* within Korean limestone regions.

#### 2.2.1. Environmental Data and Preprocessing

- In environmental data preprocessing, all predictor variables were aligned and resampled to a common grid resolution and projection (continuous variables: bilinear interpolation; categorical variables: nearest neighbour). All continuous predictors were z-score standardized prior to model fitting.
- Topographical variables were derived from the Digital Elevation Model (DEM) of the National Geographic Information Institute (NGII) [23]: (1) slope; (2) eastness and northness (aspect components); (3) terrain ruggedness index (TRI 3 × 3); (4) topographical position index (TPI) at 300 m and 900 m scales [24].
- Soil variables (CEC, clay/sand/silt content, SOC) were extracted from the 0–5 cm layer of ISRIC SoilGrids v2.0.
- Climate variables were based on observation-driven gridded products from the Korea Meteorological Administration (KMA) [25]. We used daily KMA gridded observation products and derived 10-year climatologies (2015–2024) after bias correction. Bias correction was implemented via empirical quantile mapping at each grid cell, using station observations as the reference distribution. For temperature and relative humidity, we applied additive corrections to the empirical quantiles; for precipitation, we used multiplicative scaling of non-zero daily totals, following standard practice in regional climate-impact studies. From the bias-corrected daily series, annual means (temperature, relative humidity) and annual totals (precipitation) were calculated after time-series quality control. From these fields, we derived annual mean temperature (tmean_ann), annual maximum temperature (tmax_ann), annual total precipitation (precip_ann_mm), and annual mean relative humidity (rh_ann_pct), which were used as climatic predictors in the SDM.

#### 2.2.2. Modeling Procedure and Validation

Because the Korean *C. integerrimus* population is confined to limestone areas, we first restricted the prediction domain to grid cells where limestone is present (limestone_bin = 1) and excluded this mask variable from the predictors. To prevent sparse elevation extrapolation, we further limited the prediction area to 576–1289 m a.s.l. by applying a −100 m/+200 m buffer to the empirical elevation range between the 5th and 95th percentiles (P5–P95) of the occurrence elevations.

To reduce collinearity among predictors, we computed a Spearman correlation matrix for all continuous variables and iteratively removed variables involved in multiple highly correlated pairs (|ρ| > 0.7), while always retaining elevation as a protected predictor; the resulting keep/drop decisions are summarized in Table A1. Background points were randomly stratified between the survey region (a 3 km buffer around occurrence points) and the entire limestone mask (30% buffer:70% mask). The total number of background points was adaptively set according to the number of occurrences (n < 10 → 5000; otherwise → 10,000 [26]).

The main modeling algorithm was weighted ridge logistic regression (glmnet, α = 0 [27]), with positive and negative sample weights normalized to 1:1 to mitigate class imbalance. Regularization strength (λ) was selected via internal cross-validation within glmnet. If ridge fitting failed, we used alternative logit-scale models—Maxent (maxnet) [28], bias-reduced GLM, or weighted GLM—as fall-back options. All remaining predictor variables after correlation filtering were included in the final modeling step. We selected ridge logistic regression as the primary algorithm for three reasons: (i) the number of occurrences (n = 6) is very small relative to the number of predictors, making unregularized generalized linear models highly unstable; (ii) ridge regularization is well suited to handling residual collinearity among continuous environmental predictors while retaining all variables in the model, avoiding the aggressive coefficient shrinkage and variable drop-out that can occur with lasso penalties in very small samples; (iii) in exploratory trials, ridge models produced spatial patterns qualitatively consistent with Maxent and bias-reduced GLM fits but with simpler response curves and more conservative extrapolation. Maxent, bias-reduced GLM, and weighted GLM were therefore treated as secondary, back-up models rather than primary algorithms.

After model fitting, continuous suitability maps (0–1 scale) were generated over the limestone-and-elevation mask. The primary binary threshold was defined as the upper decile (top 10%) of suitability values within the mask (area-based management target). Two correction rules—P10 (10th percentile training-presence threshold) and MTP (minimum training presence)—were subsequently applied to ensure that all occurrences were retained within the predicted suitable area, thereby determining the final threshold.

Spatial cross-validation was implemented as a simple spatial k-fold partition using k-means clustering on the projected coordinates of presence and background points. The number of folds was set adaptively to min(5, max(2, floor(n_p_/2))); for the present data (n_p_ = 6), which yielded three spatial folds. For each fold, we refitted the model on the training subset and computed the AUC and TSS (based on the Youden threshold) on the held-out test subset. We report fold-specific metrics together with summary statistics (mean ± standard deviation and median). Folds with sparse test occurrences (n_p_ < 2) were flagged and interpreted cautiously, and potential environmental extrapolation beyond the calibration space was noted.

For the final ridge model, partial dependence plots (response curves) were generated for each predictor to visually assess ecological plausibility, including the direction (sign) of effects, monotonicity, and approximate threshold ranges.

All statistical analyses were conducted in R 4.3.3 [29]. Raster processing and spatial masking/extraction were performed with terra (version 1.8-73) [30] and sf (version 1.0-23) [31]; data wrangling and table manipulation with dplyr (version 1.1.4) [32]; species distribution models were fitted with glmnet (version 4.1-10, ridge logistic regression) [27] and maxnet (MaxEnt-type exploratory models, version 4.1.4) [28]; model evaluation (AUC, TSS, and threshold calculation) used dismo (version 1.3-15) [33]; and plots and response curves were produced with ggplot2 (version 3.4.4) [34]. Final cartographic layouts and map compositions for the suitability and binary maps were prepared in ArcGIS Pro version 3.1.0. (Esri, Redlands, CA, USA [35]).

## 3. Results

### 3.1. Habitat Characteristics of Cotoneaster integerrimus in Korea

Six natural habitat sites of *Cotoneaster integerrimus* were documented in limestone areas. The habitats were broadly classified into two types: one calcareous scree site and five deciduous broad-leaved forest sites (Table 1).

In Korea, no individuals were observed on cliffs; instead, they predominantly occurred as components of the shrub layer within deciduous broad-leaved forests dominated by *Quercus mongolica*. This indicates an environment characterized by a closed overstory canopy. These five forest populations were situated under partial shade conditions, receiving filtered light through the overstory canopy (Table 1). The observed number of individuals ranged from a minimum of 5 at Site 2 to a maximum of 70 at Site 3 (Table 1).

Elevation ranged from 652 to 1150 m (median = 1043.5 m; mean = 978 m). Except for one site (site 2, 652 m), all populations showed a pronounced tendency to occur in high-elevation zones above 900 m. Among the topographic characteristics, a distinct preference for northerly aspects was observed. Except for one site (WNW, 293°), all five sites were located on north- to northeast-facing slopes.

**Figure 2:**
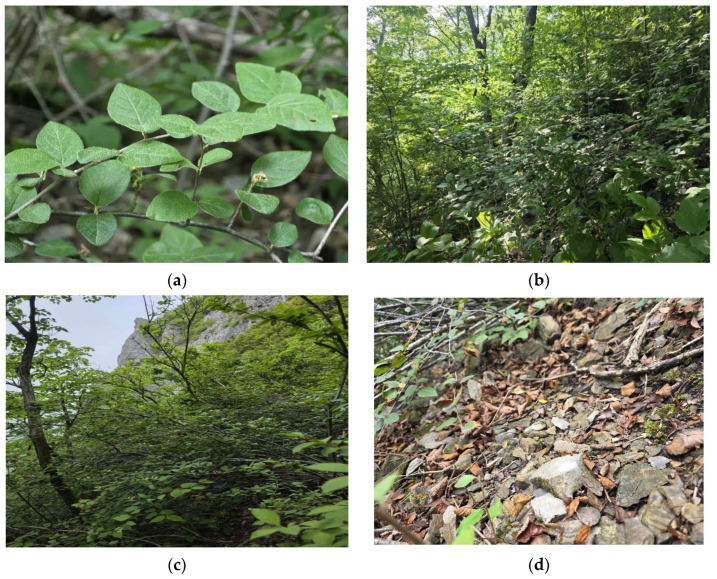
Habit and habitat of *Cotoneaster integerrimus*: (**a**) Appearance of an individual in its habitat; (**b**) Habitat in a deciduous broad-leaved forest under partial shade; (**c**) Habitat on a calcareous scree under open, full-sun conditions; (**d**) Weathered limestone scree covering the ground surface of the habitat.

The habitat slope ranged from 22° to 45° (median = 32.5°; mean = 33.7°), indicative of steep terrain; Site 1 exhibited the steepest incline (45°). Both habitat types were characterized by shallow soil profiles with abundant limestone rock fragments (debris) derived from the parent material and distributed across the soil surface and within the soil layer (Figure 2). These soils are classified as Entisols under the USDA soil taxonomy [36] and are characterized by rudimentary profile development.

In summary, *C. integerrimus* in Korea appears to be confined to a narrow ecological niche defined by a combination of edaphic properties derived from limestone parent material and montane microtopographic factors. Specifically, the habitats exhibited a distinct set of conditions: (1) alkaline, base-rich soils derived from limestone; (2) shallow, well-drained soil profiles; (3) high-elevation zones (≥900 m); (4) steep, north-facing slopes.

Given the limited number of occurrences (n = 6), model outcomes should be interpreted as preliminary and spatially conservative. Future sampling and model recalibration are required to improve statistical robustness and reduce extrapolation uncertainty.

### 3.2. Habitat Suitability Analysis for Cotoneaster integerrimus

**Figure 3:**
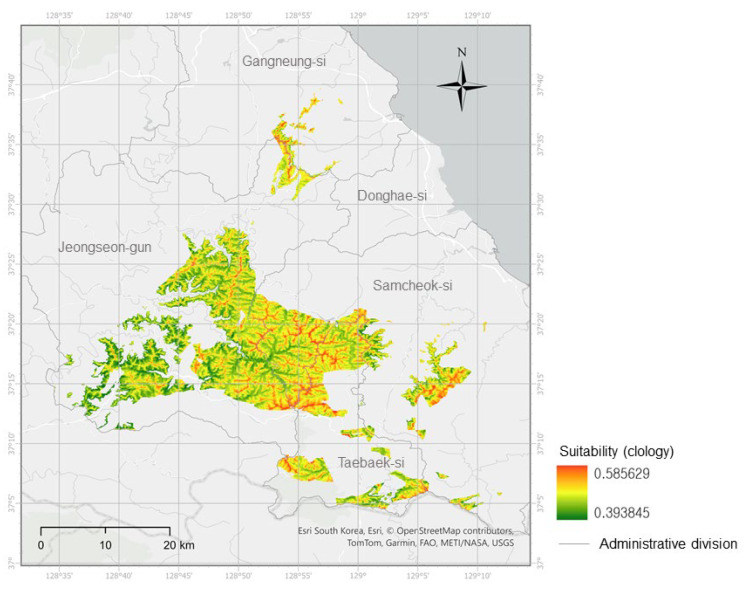
Habitat suitability map of *Cotoneaster integerrimus* (ridge-regularized logistic model) within the limestone ridge belt of central–eastern Gangwon Province (Jeongseon-gun, Samcheok-si, Taebaek-si, Donghae-si, and Gangneung-si), Republic of Korea. See Figure 1 for the national-scale location. The map is shown at the analytical raster resolution of ~90 m.

**Figure A1:**
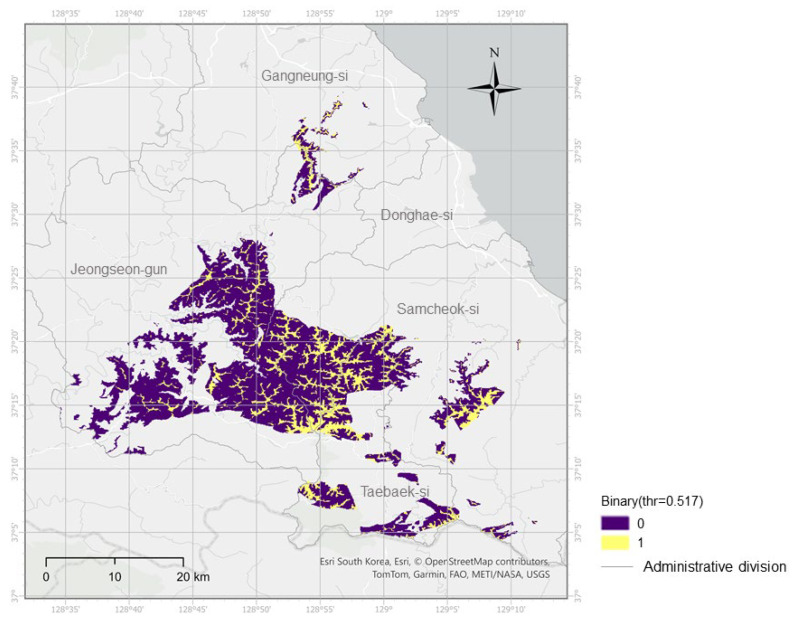
Binary habitat suitability for *Cotoneaster integerrimus* (final threshold = 0.517; spec–sens crossover = 0.503). Cells are classified as suitable (yellow) or unsuitable (purple) based on the top ~10% area criterion. The map is shown at the analytical raster resolution of ~90 m.

The final predictive model selected was ridge logistic regression (glmnet-ridge; presence/background weight = 0.5:0.5), which achieved an AUC of 0.784 on the full training dataset (Figure 3). In this training evaluation, the threshold at which sensitivity equaled specificity (spec_sens) was 0.503. For management-oriented mapping, the final threshold was defined by the upper decile (top 10%) of suitability values within the limestone mask, with additional checks using the P10 (10th percentile training presence) and minimum training presence (MTP) rules to ensure that all occurrences were retained. This yielded a final threshold of 0.517. At this threshold, occurrence coverage was 66.7%, and the proportion of positive cells in the binary map accounted for approximately 10% of the masked area (Figure A1). The binarized habitat suitability map (thr = 0.517) showed pronounced suitability along ridges and transitional slope zones, whereas suitability was low in low-elevation flat areas. A relatively continuous core area was observed in the central–western region, with satellite patches toward the northeast and southeast. These patterns suggest that targeted surveys along ridge axes would have the highest probability of discovering new populations, while the small satellite patches may be at elevated risk of local extinction and thus require careful microhabitat protection.

Spatial cross-validation using simple spatial k-fold partitioning (k-means clustering on projected coordinates; three folds for n_p_ = 6; see Section 2.2.2) revealed substantial fold-to-fold variability in performance (Table 2). Fold-wise AUC values were 0.886, 0.500, and 0.602, with corresponding TSS values of 0.886, 0.000, and 0.472. The summary statistics were AUC = 0.663 ± 0.200 (median = 0.602) and TSS = 0.453 ± 0.443 (median = 0.472). Fold 1 contained only a single test presence (nP = 1), and Fold 2 showed essentially random discrimination (AUC = 0.5, TSS = 0), illustrating the instability that arises from extremely small sample sizes and spatial clustering. Consequently, these cross-validation metrics should be interpreted as qualitative indicators of limited but non-zero discriminatory ability, rather than as precise estimates of predictive performance.

**Figure 4:**
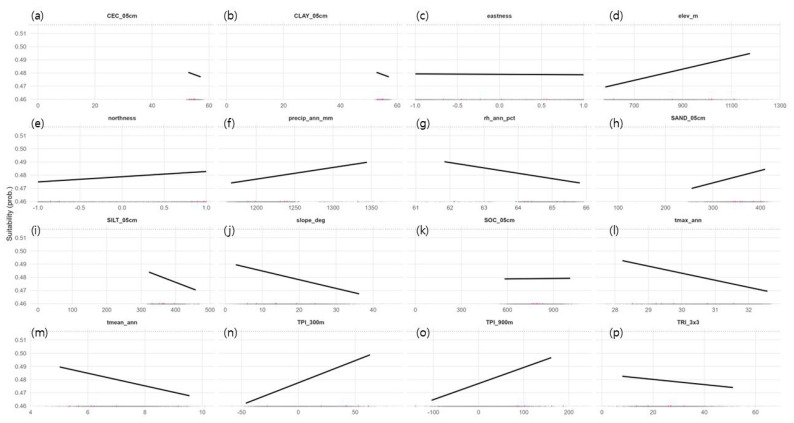
Response curves of environmental predictors (partial dependence; background-averaged). Panels: (**a**) CEC_05cm (slight –), (**b**) CLAY_05cm (−), (**c**) eastness (near-flat), (**d**) elev_m (+), (**e**) northness (+), (**f**) precip_ann_mm (+), (**g**) rh_ann_pct (−), (**h**) SAND_ 05cm (+), (**i**) SILT_05cm (−), (**j**) slope_deg (−), (**k**) SOC_05cm (near-flat), (**l**) tmax_ann (−), (**m**) tmean_ann (−), (**n**) TPI_300m (+), (**o**) TPI_900m (+), (**p**) TRI_3x3 (−).

Continuous suitability values were consistently high along ridges and slope-transition zones but low in low-lying flatlands. According to the partial dependence plots (conditional predictions at the background mean), elevation (elev_m) and TPI (300–900 m) showed positive (+) effects, whereas slope (slope_deg) and terrain ruggedness (TRI_3x3) showed negative (−) effects (Figure 4). Among climatic variables, annual mean temperature (tmean_ann) and annual maximum temperature (tmax_ann) had negative (−) effects, while annual precipitation (precip_ann_mm) exhibited a mild positive (+) trend. Aspect variables indicated a weak positive (+) effect of northness, whereas eastness had little influence. For soil variables, topsoil sand content (SAND_05cm) showed a weak positive (+) tendency, while silt and clay contents displayed weak negative (−) trends. These patterns are consistent with the well-drained, shallow soils and gravel- to rock-rich conditions observed in limestone ridge habitats during field surveys. However, the reliability of these effect estimates should be interpreted with caution, given both the coarse resolution of the SoilGrids dataset and the small sample size. Overall, the response curves should be read as qualitative tendencies within a cool limestone ridge syndrome, rather than as precise, independent effects of individual predictors.

## 4. Discussion

### 4.1. Ecological Specificity and Conservation of Native Korean Habitats

A common characteristic of *Cotoneaster integerrimus* habitats in both Korea and Europe is their restriction to limestone regions [37]. Soils derived from limestone are typically alkaline (pH > 7) [38], creating a distinctive substrate for calcicolous plants [39,40]. Consequently, these regions harbor unique vegetation and hold substantial ecological conservation value [41].

In Europe, *C. integerrimus* is a diagnostic, heliophilous species of open limestone shrub communities [42,43,44,45,46], preferring areas with high light availability [47]. In contrast, Korean populations predominantly inhabit the forest understory, a divergence likely driven by distinct climatic conditions. The hot, humid Korean summers (mean ~25 °C) contrast sharply with the cooler European conditions (7–21 °C) [48]. In rocky environments, such high temperatures induce severe drought stress [49]. Thus, the preference of Korean populations for semi-shaded, north-facing slopes likely represents an adaptive strategy to mitigate heat and maintain soil moisture. These findings suggest that, unlike their European counterparts, Korean populations have undergone local adaptation to the temperate monsoon climate and episodic drought stress.

The distribution of *C. integerrimus* appears strongly thermally limited. While European populations exhibit diverse vertical distributions depending on latitude [6,8], Korean populations are confined to high elevations. Biogeographically, widespread Eurasian populations likely experienced range contractions during Pleistocene climatic oscillations [50]. Consequently, the high-elevation Korean populations may represent glacial relicts that persisted in favorable microclimatic zones.

The Korean population represents a Geographically Peripheral Population (GPP) at the species’ easternmost limit. Such populations are critical for conservation as they often harbor unique genotypes [51,52]. Having likely diverged ecologically due to long-term isolation, the Korean population holds substantial value for maintaining the species’ genetic diversity. However, it faces dual threats from small population size and habitat fragmentation. Furthermore, global warming is projected to further shrink its potential habitat, placing these GPPs at high risk [53,54]. Therefore, active conservation strategies integrating in situ and ex situ approaches are urgently needed to safeguard this unique genetic resource.

Although population-level genetic data are currently unavailable, the observed ecological and morphological divergence strongly supports regional differentiation. Future molecular analyses will be essential to validate these findings and refine conservation strategies.

### 4.2. Ecological Interpretation of the Model, Validation Constraints, and Conservation Implications Under Climate Change

Given the very limited number of occurrences (n = 6), the SDM results should be regarded as preliminary and spatially conservative. The ridge logistic regression model achieved a moderate training AUC of 0.784, but spatial k-fold cross-validation with three k-means folds (Table 2) showed strong variability, including one fold with essentially random discrimination (AUC = 0.5, TSS = 0) and another with a single test presence (n_p_ = 1). We therefore use the model as a screening tool to highlight candidate survey areas, rather than as a definitive basis for fine-scale mapping or regulatory zoning. Additional field surveys, including both new presences and structured background/absence data, are planned to improve statistical robustness and reduce extrapolation uncertainty.

Within this cautious framework, the main ecological signals are broadly consistent with field observations. Elevation and ridge position (TPI at 300–900 m) increase predicted suitability, whereas slope, terrain ruggedness, and high-temperature variables have negative effects, matching the concentration of occurrences along ridge-to-slope transition zones. Aspect appears secondary: northness shows only a weak positive tendency, while eastness has little influence. The negative grid-scale effects of slope and TRI likely reflect the limited resolution of the DEM, which cannot fully capture fine-scale microtopography (e.g., small scarps, crevices) and associated shading and moisture retention; future re-validation that incorporates microclimatic predictors (e.g., heat-load or shade indices and soil-moisture metrics) would help refine these interpretations.

The cross-validation results further emphasize the uncertainty that arises from a very small and spatially clustered sample. In this context, the fold-wise metrics are best viewed as descriptors of instability rather than precise performance estimates, and the median across folds provides a more robust summary than the mean. We retain all fold-specific values in Table 2 to transparently document these limitations for conservation and policy use.

For management, the final threshold of 0.517 represents a pragmatic, management-oriented cutoff that selects the top 10% of suitability values within the limestone mask while retaining 66.7% of known occurrences. We recommend that conservation planning focus primarily on the continuous suitability surface, using the binary map only as a communication aid and a guide for follow-up field checks. The elevation mask (576–1289 m), defined by extending the observed elevation range (P5–P95) by –100/+200 m, helps avoid unrealistic extrapolation into lowlands. However, because elevation and temperature are partly confounded in the study area, the positive effects of elevation and TPI and the negative effects of mean and maximum temperature are best interpreted as a joint “cool limestone ridge” syndrome rather than independent causal gradients. Even after correlation filtering and ridge regularization, residual collinearity remains, so we treat the response curves as qualitative summaries of this habitat syndrome, not as estimates of isolated predictor effects.

Finally, as a potential glacial relict at the eastern edge of the species’ range, the Korean *C. integerrimus* population is likely to be sensitive to interannual variability and long-term warming. A logical next step will be to project the current ridge model under CMIP6 climate scenarios (e.g., SSP1–2.6, SSP2–4.5, SSP5–8.5) to identify zones of climatic extrapolation, quantify projection uncertainty, and locate climatically stable refugia. Any such projections will need to be interpreted with the same caution as the present model—explicitly acknowledging the small sample size and residual collinearity—but they could nonetheless provide a useful empirical basis for prioritizing conservation actions and anticipating climate-driven habitat shifts.

### 4.3. Geographical Disjunction and Morphological Variation in the Korean Cotoneaster integerrimus

The genus Cotoneaster is widely recognized as a taxonomically intricate group, largely due to high intraspecific variation arising from polyploidy and apomixis [55,56,57]. These reproductive mechanisms generate extensive morphological variation and clinal continua. *C. integerrimus* is broadly distributed across Europe and Asia, and its populations occur in geographically disjunct and isolated habitats, raising questions about the extent of morphological variation associated with these geographic extremes. Based on morphological variation, several researchers have attempted to classify specific regional populations as distinct species. For instance, Fryer and Hylmö described *C. cambricus* [58], and Gandoger proposed *C. pyrenaicus* [59]. In contrast, Dickoré and Kasperek criticized these taxonomic separations for disregarding the continuous nature of morphological variation and instead advocated for a broader species concept (*C. integerrimus sensu lato*) encompassing numerous micropopulations [2]. Although recent advances in genomic analysis have facilitated more comprehensive phylogenetic assessments, direct morphological comparisons between East Asian and European populations—situated at the opposite extremes of the Eurasian continent—remain lacking.

**Figure 5:**
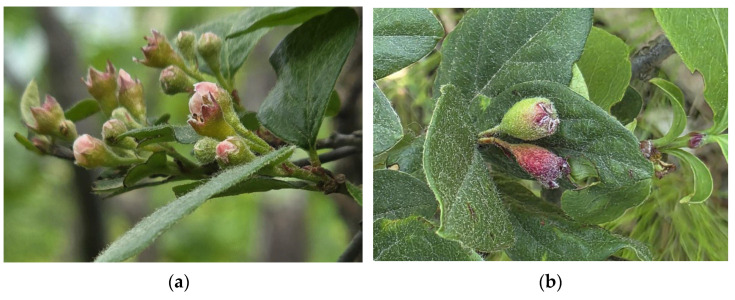
Key morphological characteristics of Korean *Cotoneaster integerrimus*: (**a**) pubescent calyx and (**b**) pubescent fruit.

The Korean population examined in this study exhibits distinctive morphological and ecological traits that are absent in European [7] counterparts. Notably, the Korean population displays a densely hairy calyx, with trichomes persisting until seed maturation (Figure 5)—a diagnostic feature not documented in European material [60]. Such long-term geographical disjunction aligns with the fixation of unique phenotypic traits within the isolated Korean population. This observed morphological distinctiveness is consistent with that the East Asian lineage having developed unique characteristics distinguishable from European populations [61,62].

From an ecological perspective, the preference of the Korean population for forest interiors, semi-shaded microhabitats, and north-facing slopes indicates adaptation to microclimatic conditions that mitigate heat and humidity during the summer season. Such habitat preference represents an adaptive strategy to the temperate monsoon climate, suggesting that the Korean population exhibits ecological characteristics consistent with local adaptation facilitated by geographic isolation and regional climatic conditions.

In conclusion, the population isolated on the Korean Peninsula displays unique morphological and ecological profiles, likely shaped by geographic isolation and environmental conditions distinct from those of European populations. To rigorously determine whether these variations reflect genetic differentiation, further research is required, including (i) common-garden and reciprocal transplant experiments to distinguish phenotypic plasticity from local adaptation and (ii) genomic analyses to quantify genetic divergence and infer gene flow. Accumulation of such evidence will allow for a clearer understanding of the evolutionary distinctiveness and taxonomic status of the Korean population.

## 5. Conclusions

This study aimed to elucidate the ecological characteristics of the rare plant *Cotoneaster integerrimus*, isolated at the easternmost limit of the Eurasian continent, and to predict its potential habitat distribution using Species Distribution Modeling (SDM).

The results indicate that the Korean population of *C. integerrimus* exhibits distinct ecological characteristics that clearly differentiate it from European populations. Unlike European populations, which are heliophilous and typically inhabit open limestone rock areas, the Korean population showed a preference for partial shade and north-facing slopes within the forest understory. This distribution pattern is interpreted as an adaptive strategy to mitigate high-temperature and drought stress under the hot and humid temperate monsoon climate of the Korean Peninsula.

Moreover, a unique morphological trait not previously reported in European populations was observed: the presence of dense, persistent calyx hairs that remain until fruit maturity. The combination of these ecological and morphological divergences supports the hypothesis of local adaptation facilitated by geographic isolation.

The SDM results were broadly consistent with these field observations, highlighting high elevation as a key determinant of suitable habitat. Conversely, high-temperature variables, such as annual mean temperature, emerged as primary limiting factors reducing habitat suitability.

Despite the limitations of a six-point SDM, the broad-scale patterns emerging from our analyses provide a useful first approximation of where cool, limestone ridge habitats for *C. integerrimus* are most likely to occur in South Korea. These findings have important implications. Firstly, as a potential glacial relict and a GPP, the Korean *C. integerrimus* population is of high conservation priority for maintaining the species’ genetic diversity. Secondly, this temperature-sensitive population is highly vulnerable to climate change and faces compounded risks associated with small population size and habitat fragmentation.

Consequently, our study supports the view that the Korean population, situated at the easternmost limit of the species’ Eurasian range, has acquired distinct traits through long-term geographic isolation. Uncovering the evolutionary distinctiveness of this peripheral population provides a crucial baseline for future research. Therefore, to safeguard these unique genetic resources and anticipate climate-driven shifts, further research is urgently needed, including projections under future climate scenarios, establishment of ex situ conservation strategies, genomic analyses, and common-garden experiments.

## Figures and Tables

**Table 1:** Environmental characteristics of the habitat of *Cotoneaster integerrimus* in Korea (H.T.: Habitat type, Al.: Altitude, N.I.: Number of individuals, A.C.: Area covered, As.: Aspect, S.A.: Slope angle, L.C.: Light condition).

Site	H.T.	Al. (m)	N.I.	A.C. (m^2^)	As. (°)	S.A. (°)	L.C.
1	Calcareous scree	1037	30	80	WNW (293)	45	Full sun
2	Deciduous broad-leaved forest	652	5	25	NE (45)	40	Partial shade
3	Deciduous broad-leaved forest	1150	70	110	N (0)	30	Partial shade
4	Deciduous broad-leaved forest	1050	30	80	NNE (23)	35	Partial shade
5	Deciduous broad-leaved forest	922	60	120	N (355)	22	Partial shade
6	Deciduous broad-leaved forest	1057	50	96	NE (48)	30	Partial shade

**Table 2:** Spatial k-fold cross-validation (k-means clustering, k = 3) performance by fold (AUC, TSS) with summary statistics.

Fold	AUC	Thr_Spec_Sens	TSS	nP	nB
1	0.886	0.498	0.886	1	1272
2	0.500	0.632	0.000	2	1161
3	0.602	0.493	0.472	3	2561

## Data Availability

Primary data (location coordinates) and analysis resources are available from the corresponding author on reasonable request under a data-use agreement (non-commercial research/conservation only; no redistribution of coordinates; citation required; data destruction at project end) and, where applicable, approval by the competent authority. Requests should be emailed to the corresponding author with a brief project description and confirmation of the above conditions.

## Appendix A

**Table A1:** Collinearity filtering results for environmental predictors. For each variable, kept indicates whether it was retained in the final model, protected identifies predictors that were always retained (elevation), and closest_kept shows the most strongly correlated retained variable for each dropped predictor. Only tmin_ann was removed due to high correlation with rh_ann_pct. NA indicates that no closest kept variable is applicable because the predictor was retained in the final model.

Var	Kept	Protected	Closest_Kept
elev_m	TRUE	TRUE	NA
slope_deg	TRUE	FALSE	NA
eastness	TRUE	FALSE	NA
northness	TRUE	FALSE	NA
TRI_3x3	TRUE	FALSE	NA
TPI_300m	TRUE	FALSE	NA
TPI_900m	TRUE	FALSE	NA
CEC_05cm	TRUE	FALSE	NA
CLAY_05cm	TRUE	FALSE	NA
SAND_05cm	TRUE	FALSE	NA
SILT_05cm	TRUE	FALSE	NA
SOC_05cm	TRUE	FALSE	NA
tmean_ann	TRUE	FALSE	NA
tmin_ann	FALSE	FALSE	rh_ann_pct
tmax_ann	TRUE	FALSE	NA
precip_ann_mm	TRUE	FALSE	NA
rh_ann_pct	TRUE	FALSE	NA

## References

[1] Cumming W.A. (1960). Germination studies with *Cotoneaster lucida* being conducted at the Canada Experimental Farm, Morden, Manitoba. West. Can. Soc. Hortic. Rep. Proc..

[2] Dickoré W.B., Kasperek G. (2010). Species of *Cotoneaster* (*Rosaceae*, *Maloideae*) indigenous to, naturalising or commonly cultivated in Central Europe. Willdenowia.

[3] Kicel A. (2020). An Overview of the Genus *Cotoneaster* (Rosaceae): Phytochemistry, Biological Activity, and Toxicology. Antioxidants.

[4] Bogunić F., Siljak-Yakovlev S., Mahmutović-Dizdarević I., Hajrudinović-Bogunić A., Bourge M., Brown S.C., Muratović E. (2021). Genome Size, Cytotype Diversity and Reproductive Mode Variation of *Cotoneaster integerrimus* (Rosaceae) from the Balkans. Plants.

[5] Boratyński A., Kosiński P., Kwiatkowski P., Szcześniak E., Świerkosz K. (1999). Protected and deserving protection trees and shrubs of the Polish Sudety Mts. with their pre-alps. 11: *Cotoneaster integerrimus* Medik. and *Cotoneaster niger* (Thunb.) Fr. Arbor. Korn..

[6] Blamey M., Grey-Wilson C. (1989). Flora of Britain and Northern Europe.

[7] Tutin T.G., Heywood V.H., Burges N.A., Moore D.M., Valentine D.H., Walters S.M., Webb D.A. (1968). Flora Europaea, Vol. 2: Rosaceae to Umbelliferae.

[8] Świerkosz K., Reczyńska K. (2022). Differentiation of natural scrub communities of the *Cotoneastro-Amelanchieretum* group in Central Europe. PLoS ONE.

[9] European Environment Agency Rock Pear Scrub (EUNIS Habitat Type F3.1123/S3-5123). EUNIS Habitat Classification. https://eunis.eea.europa.eu/habitats/22561.

[10] Punegov A.N., Skrotskaya O.V., Nikolaevna S.A. (2024). Introduction of species of the genus *Cotoneaster* Medik. in the changing climate of the Komi Republic. Samara J. Sci..

[11] Lomolino M.V., Riddle B.R., Whittaker R.J. (2016). Biogeography.

[12] Primack R.B. (2014). A Primer of Conservation Biology.

[13] Ravanbakhsh H., Hamzeh’ee B., Moshki A. (2018). Ecology and phytosociology of *Cotoneaster* shrublands in Central Alborz of Iran. Dendrobiology.

[14] Lu L.D., Brach A.R., Wu Z.Y., Raven P.H., Hong D.Y. (2003). *Cotoneaster* Medik. Flora of China, Vol. 9: Pittosporaceae Through Connaraceae.

[15] Angmo D., Puri R., Mehta M., Devi G. (2022). Ethnobotanical survey of wild edible plants of Leh District, Ladakh. Def. Life Sci. J..

[16] Choi Y.M., Cho S.J., Lee H.J., Yoon J.W. (2024). A Checklist of North Korea Plant and Current Status of Genetic Resources Held by Domestic and International Arboreta. Korean J. Plant Res..

[17] Komarov V.L. (1901). Flora Manshuriae.

[18] Yang J., Park S., Kim Y., Kim J.S., Pak J.-H. (2022). Infrageneric plastid genomes of *Cotoneaster* (Rosaceae): Implications for the plastome evolution and origin of *C. wilsonii* on Ulleung Island. Genes.

[19] Guo X., Bai W., Wang Y., Hao S., Zhao L., Li X., Guo Z., Li X. (2025). Predicting habitat suitability for an endangered medicinal plant, *Saussurea medusa*: Insights from ensemble species distribution models. Front. Plant Sci..

[20] Augustin A.F., Lima D.F., Vieira F.C.S., Caddah M.K. (2025). Species distribution modeling of two rare endemic Myrtaceae from the Brazilian Atlantic Forest: Challenges in conserving invisible species. Flora.

[21] Onete M., Bodescu F.P., Niccoara R., Manu M., Mihailescu S. (2025). Modelling the distribution of the rare endemic, tertiary relict and vulnerable species (Western Romanian Carpathians). Sci. Pap. Ser. B Hortic..

[22] Han L., Ma X., Zhao C., Liu D. (2025). Influences of environmental and leaf functional traits variations on photosynthetic characteristics of *Cotoneaster multiflorus* in Xinglong Mountain. Front. Plant Sci..

[23] National Geographic Information Institute (NGII) Digital Elevation Model (DEM) Data. https://www.ngii.go.kr/kor/main.do.

[24] Weiss A.D. (2001). Topographic Position and Landforms Analysis; Poster Presentation, ESRI User Conference, San Diego, CA, USA. https://karttur.github.io/geoimagine/blog/blog-ArcticDemTPI/.

[25] Korea Meteorological Administration (KMA) (2020). KMA Gridded Observation Data Products.

[26] Phillips S.J., Anderson R.P., Schapire R.E. (2006). Maximum Entropy Modeling of Species Geographic Distributions. Ecol. Model..

[27] Friedman J., Hastie T., Tibshirani R. (2010). Regularization Paths for Generalized Linear Models via Coordinate Descent. J. Stat. Softw..

[28] Phillips S.J., Anderson R.P., Dudík M., Schapire R.E., Blair M.E. (2017). Opening the black box: An open-source release of Maxent. Ecography.

[29] R Core Team (2023). R: A Language and Environment for Statistical Computing.

[30] Hijmans R.J. (2025). terra: Spatial Data Analysis.

[31] Pebesma E. (2018). Simple features for R: Standardized support for spatial vector data. R J..

[32] Wickham H., François R., Henry L., Müller K., Vaughan D. (2025). dplyr: A Grammar of Data Manipulation.

[33] Hijmans R.J., Phillips S., Leathwick J., Elith J. (2024). dismo: Species Distribution Modeling.

[34] Wickham H., Bryan J. (2023). ggplot2: Elegant Graphics for Data Analysis. R Package Version 3.4.4. https://CRAN.R-project.org/package=ggplot2/.

[35] ESRI (2024). ArcGIS Pro Version 3.1.0.

[36] National Institute of Agricultural Sciences (NIAS), Rural Development Administration (RDA) Soil Data Portal. https://soil.rda.go.kr/geoweb/soilmain.do.

[37] Kurtto A., Sennikov A.N., Lampinen R. (2013). Atlas Florae Europaeae: Distribution of Vascular Plants in Europe.

[38] Brady N.C., Weil R.R. (2016). The Nature and Properties of Soils.

[39] Hwang K.S. (1973). Survey on the pH of Soils in Korea. Korean J. Soil Sci. Fertil..

[40] Craw D., Rufaut C., Palmer M. (2025). Geoecology of limestone-hosted dryland calcicolous plants, North Otago, New Zealand. N. Z. J. Geol. Geophys..

[41] Vermeulen J., Wiriadinata H. (2005). Biodiversity, Endemism and the Conservation of Limestone Karsts in the Sangkulirang Peninsula, Borneo. Trop. Biodivers. Conserv..

[42] Mucina L., Bültmann H., Dierßen K., Theurillat J.P., Raus T., Čarni A., Šumberová K., Willner W., Dengler J., Gavilán García R. (2016). Vegetation of Europe: Hierarchical floristic classification system of vascular plant, bryophyte, lichen, and algal communities. Appl. Veg. Sci..

[43] Willner W., Tichý L., Chytrý M. FloraVeg.EU: European Vegetation Database.

[44] Chytrý M., Danihelka J., Kaplan Z., Pyšek P. *Pladias: Database of the Czech Flora and Vegetation*; Institute of Botany, CAS: Průhonice, Czech Republic. https://pladias.cz/en/taxon/data/Cotoneaster%20integerrimus.

[45] InfoFlora Cotoneaster integerrimus Medik.

[46] Pignatti S., Guarino R., La Rosa M. (2017). Flora d’Italia.

[47] Chytrý M., Řezníčková M., Novotný P., Holubová D., Preislerová Z., Attorre F., Biurrun I., Blažek P., Bonari G., Borovyk D. (2024). FloraVeg.EU—An Online Database of European Vegetation, Habitats and Flora. Appl. Veg. Sci..

[48] Kim J.U., Boo J.O., Shim S.S., Kwon W.T., Byun Y.H. (2017). The Seasonal Correlation between Temperature and Precipitation over Korea and Europe and the Future Change from RCP8.5 Scenario. Atmosphere.

[49] Larcher W. (2003). Physiological Plant Ecology: Ecophysiology and Stress Physiology of Functional Groups.

[50] Lo E.Y.Y., Donoghue M.J. (2012). Expanded phylogenetic and dating analyses of the apples and their relatives (Pyreae, Rosaceae). Mol. Phylogenet. Evol..

[51] Lesica P., Allendorf F.W. (1995). When Are Peripheral Populations Valuable for Conservation?. Conserv. Biol..

[52] Eckert C.G., Samis K.E., Lougheed S.C. (2008). Genetic Variation across Species’ Geographical Ranges: The Central–Marginal Hypothesis and Beyond. Mol. Ecol..

[53] Hampe A., Petit R.J. (2005). Losing Your Edge: Climate Change and the Conservation Value of Range-Edge Populations. Ecol. Lett..

[54] IUCN SSC Climate Change Specialist Group (2007). Vital but Vulnerable: Climate Change Vulnerability and the Conservation of Species with Restricted Ranges and Small Populations.

[55] Li J., Zhang L., Liu Y., Wang H., Zhou S. (2014). Molecular Phylogeny of *Cotoneaster* (Rosaceae) Inferred from Nuclear ITS and Multiple Chloroplast Sequences. Plants.

[56] Huang J., Chen S., Meng K., Li M., Zhao W., Wang N., Fan Q., Liao W. (2025). Phylogenomic relationships and species delimitation of *Cotoneaster* ser. Pannosi, ser. Buxifolii, and related taxa. Front. Plant Sci..

[57] Kroon H. (1975). Polyploidy in Cotoneaster II. Bot. J. Linn. Soc..

[58] Fryer J., Hylmö B. (1994). The Native British *Cotoneaster*—Great Orme Berry—Renamed. Watsonia.

[59] Gandoger M. (1875). Decades Plantarum Novarum: Præsertim ad Floram Europae Spectantes.

[60] Fryer J., Hylmö B. (2009). Cotoneasters: A Comprehensive Guide to Shrubs for Flowers, Fruit, and Foliage.

[61] Dudaniec R.Y., Schlotfeldt B.E., Bertozzi T., Donnellan S.C., Kleindorfer S. (2011). Genetic and morphological divergence in island and mainland birds: Informing conservation priorities. Biol. Conserv..

[62] Zumwalde B.A., McCauley R.A., Fullinwider I.J., Duckett D., Spence E., Hoban S. (2021). Genetic, Morphological, and Environmental Differentiation of an Arid-Adapted Oak with a Disjunct Distribution. Forests.

